# Squamous cell carcinoma of esophagus in a 15-year-old boy

**DOI:** 10.4103/0971-9261.70641

**Published:** 2010

**Authors:** J. B. Hedawoo, N. G. Nagdeve, G. N. Sarve

**Affiliations:** Department of Surgery, Government Medical College, Nagpur, India

**Keywords:** Esophageal carcinoma, squamous cell carcinoma, teenage

## Abstract

A 15-year-old boy with well-differentiated squamous cell carcinoma of the lower esophagus is reported because of its rarity. The patient presented with dysphagia for 3 months and weight loss. The case was treated with radical excision, with excellent immediate response.

## INTRODUCTION

Carcinoma of the esophagus is uncommon in younger age groups[1] and extremely rare in children and adolescents, with only isolated case reports.[2–4] Herein, we report a case of squamous cell carcinoma involving the lower-third of the esophagus in a 15-year-old boy because of its rarity in the teenage group.

## CASE REPORT

**Figure 1:**
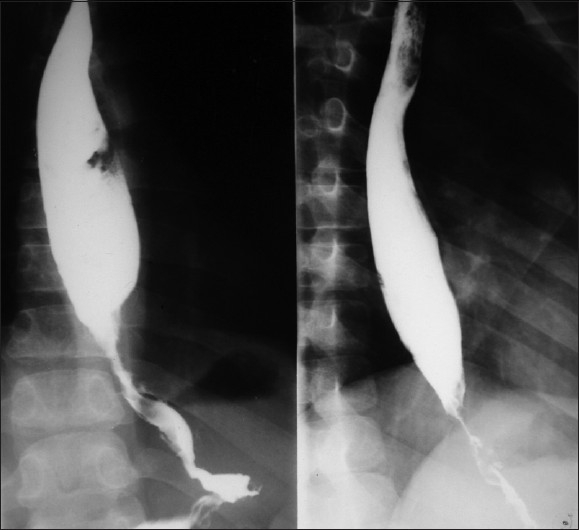
Barium swallow showing the typical rat-tail appearance

**Figure 2:**
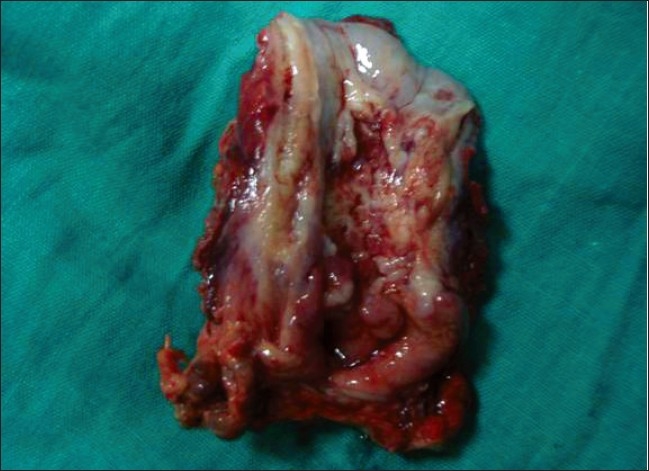
Photograph of the resected specimen of the esophagus with ulcerated growth and gastric fundus

**Figure 3:**
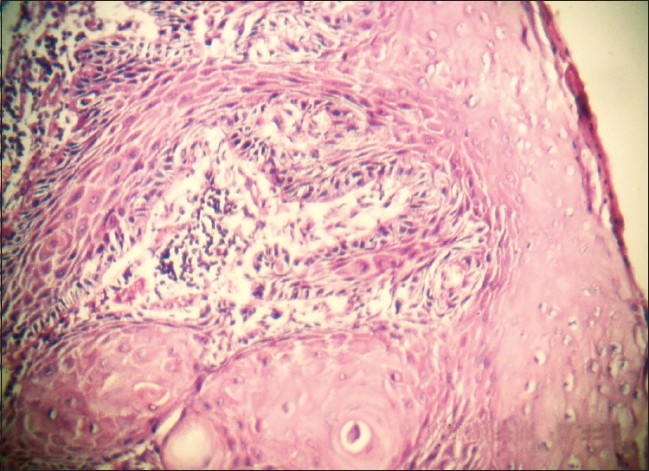
Microphotograph of the resected specimen (H&E, ×200)

A 15-year-old boy presented with dysphagia of 3 months duration, initially for solid food, which progressively increased to liquids. In the last 6 weeks, he was unable to swallow even liquids. He lost 5 kg in 2 months. There was no history of pain during deglutition, chest pain, regurgitation or vomiting. He was eating betel nut for the last 3 years. There was no history of ingestion of any corrosive substance. There was no family history of esophageal problems or gastrointestinal malignancies and no known familial or genetic disorder. Except for his thin built, systemic examination was unremarkable. There was no keratinization of his palms or soles to suggest tylosis. Except for low hemoglobin level (8.4 gm%), other laboratory investigations and chest radiographs were normal. A barium swallow showed a rat-tail appearance of the lower-third of the esophagus with normal stomach [Figure 1]. An upper gastrointestinal (GI) endoscopic examination showed an ulcerated growth at 25 cm obstructing the lumen. A biopsy from the growth revealed a well-differentiated keratinizing squamous cell carcinoma. A contrast-enhanced computed tomogram (CECT) scan of the chest and abdomen failed to reveal any distant metastases. Resection of the esophagus was planned through left thoraco-abdominal approach. At surgery, there was a large growth arising from the abdominal esophagus [Figure 2]. The growth had involved both crura of the diaphragm. Also, there was involvement of the peri-esophageal lymph nodes. En-bloc resection of the esophagus to the level of carina, gastric fundus, both diaphragmatic crura and lymph nodes was carried out. Esophago-gastric continuity was established using a circular stapler no 25. Pyloroplasty and feeding jejunostomy were performed. The postoperative period was uneventful and the patient was allowed oral feeds on the 7^th^ postoperative day. Histology of the resected specimen confirmed a well-differentiated squamous cell carcinoma with tumor-free margins [Figure 3]. Lymph nodes were free from tumor cells.

## DISCUSSION

The idea regarding rare occurrence of esophageal carcinoma in children comes from the fact that a national survey conducted in the USA during 1952–1956 reported only three deaths from esophageal tumors in the age group of 0–14 years, while a comparable series from England did not report such deaths.[5]

A search of the literature revealed only a few cases of esophageal carcinoma in children reported so far.[2–4] The youngest patient reported so far was an 8-year-old Indian girl with esophageal carcinoma in the middle third of the esophagus with lung metastases.[6] Interestingly, esophageal carcinoma in teens has been reported more frequently from India than any other part of world. More than 10 cases are reported from this part of the world.

Various studies from endemic areas suggest a predominant role for environmental factors in esophageal carcinogenesis. Studies have suggested that carcinogens may be derived from tobacco, alcohol, moldy foods or certain spices. Nutritional deficiencies such as low levels of riboflavin, retinol and zinc exposure to N-nitrosamines and fungal contamination of foods may also be associated with the disease. The prevalence of esophageal carcinoma is high in siblings with esophagitis and in patients with a family history of esophageal cancer. Tylosis, achalasia, esophageal diverticula, lye stricture and Plummer–Vinson syndrome are considered as premalignant conditions for esophageal carcinoma. But, none of these conditions have been reported to cause the esophageal carcinoma at teenage. There is a report of a 15-year-old boy with well-differentiated squamous cell carcinoma of the upper esophagus with a significant past history of accidental lye ingestion.[7] However, studies have shown that a long latency period (12 years or more) is required between the ingestion of lye and the development of the malignancy. Also, there is a report of human papillomavirus-associated esophageal carcinoma from India.[8] In our patient, there was a history of chewing of betel nut for 3 years, but its significance in causation of malignancy is questionable. Also, we could not find any familial risk factors or premalignant conditions.

The rarity of esophageal malignancies in children may be due to the fact that these tumors are predominantly produced by the environment, which requires long latent periods of at least 15–20 years to manifest as carcinomas. Exposures to environmental risk factors and nutritional deficiencies in childhood and early life might be responsible for inflammation and weakened esophageal epithelium, resulting in a condition possibly more favorable for the development of esophageal cancer.

The management of the disease depends on the stage, and equally good results are reported with surgery and radiotherapy for operable disease. Also, the outcome depends on the stage of the disease and is not influenced by age. Our patient is enjoying excellent health and there is no evidence of recurrence. To conclude, although rare, esophageal carcinoma must be considered in the differential diagnosis of dysphagia even at an early age.
